# Abstract of a Paper on the Influence of Alcoholic Drinks, on the Development and Progress of Tuberculosis

**Published:** 1861-02

**Authors:** N. S. Davis


THE CHICAGO
MEDICAL EXAMINER

VOL. II. FEBRUARY 1861. No. 2

### ORIGINAL CONTRIBUTIONS.

---

*Abstract of a Paper on the Influence of Alcoholic Drinks, on the Development and Progress of Tubercular Diseases, and especially Pulmonary Tuberculosis; read to the Section of Practical Medicine and Obstetrics, during the Session of the American Medical Association for* 1860. New Haven, Oct. June 5th, 1860. By N. S. DAVIS, M. D.

When we remember that about *one seventh* of all the deaths in our country, and also in some of the more enlightened nations of Europe, are produced by tubercular disease of the Lungs, we shall readily see the importance of investigating closely and critically the influence of every agent that may be supposed either to favor or retard its development.

For many generations, alcoholic drinks have been so generally in use by all classes of people, as to exert an important influence for good or evil, on the health of individuals and communities. It was not, however, until within the last six or eight years that the idea was announced, and rapidly circulated through the various periodicals of the country, that alcoholic beverages, and especially genuine Bourbon Whiskey and pure Lager Beer, were capable of, both preventing the development of pulmonary tuberculosis, and of retarding its progress after it had been developed. This announcement not being in consonance with many facts that had previously come under my observation, led me to commence immediately a record of all well marked cases of Tuberculosis that came under my care, and from the subjects of which I could obtain a perfectly reliable account of their previous habits in relation to the use of alcoholic beverages, whether fermented or distilled.

The result of my observations for the first year were reported to the Illinois State Medical Society, at its annual meeting in June, 1856. Since that time the record has been extended until it embraces 210 cases, including the records of both hospital and private practice. Of these cases 140 were males, and 70 females. Eighty-five were natives of Ireland; 60 of the United States; 25 of Norway and Sweeden; 20 of Germany; 15 of England, Scotland and Wales. To prevent erroneous conclusions in regard to these statistics of nativity, we should state that in Chicago, where these observations were made, about one half of the entire population are of foreign birth. And of this half, far the greater part are from Ireland and the German States, though both Swedes and Norwegians are numerous. These statistics being gathered from my own personal observations in practice, it is proper to state that through my connection with the Hospital of the Sisters of Mercy, I come in practical contact with a much larger population of the natives of Ireland than of any other class of foreigners. This will account in *part* for the very large relative proportion of cases from that class of people. The leading object in recording all these cases, was to ascertain just how far the subjects of them had been under the influence of alcoholic drinks, for a period of not less than one year previous to any active or noticable symptoms of the tuberculous disease.

To show the results in reference to this point, I have been obliged to divide the whole number into three classes. The first, were such as had used some form of alcoholic beverage almost daily from one to twelve years previous to the active signs of tuberculosis; the second, such as used these drinks occasionally; and the third, such as had wholly abstained from their use.

Of the 210 cases, 68 belonged to the first class; 91 to the second; and 51 to the third. Although so large a proportion as 68 out of the 210 cases, were habitual drinkers of alcoholic liquors, only 15 of that number were such as are usually called *drunkards.* Five of these were admitted into the Hospital while uffected with delirium tremens, and also in the advanced stage of tubercular phthisis. Among the 53 cases occurring in those habitually using alcoholic beverages, yet not to the extent of producing drunkenness, there were many presenting circumstances as favorable for determining the question whether these agents are capable of preventing the development of pulmonary Tuberculosis, as though they had been selected by a judicious practitioner purposely for an experiment lasting through a series of years. For instance, a man aged about 45 years, native of Ireland; had resided in the State of Wisconsin for the last twelve years, keeping a public house or tavern in a healthy rural district. He was an intelligent and candid man, and had come to Chicago to consult me in relation to his health. I found him much emaciated, with hectic fever, purulent expectoration, and the physical signs of cavities in the upper lobes of both lungs, surrounded by tuberculous deposits in different stages of advancement. In answer to my inquiries, he said his cough and first symptoms of disease had commenced gradually, about eighteen months previously—had lived in a country district twelve years, during which time, though keeping a *Tavern*, he had taken a fair degree of out-door exercise—had generally enjoyed good health—and had been in the habit, during the whole time, of taking from one to three or four moderate drinks of alcoholic liquors every day, (chiefly Brandy and Whiskey), but had never drank to the extent of producing ordinary intoxication. He denied all knowledge of any hereditary tendency to consumption in the family to which he belonged. This case terminated fatally in about two months after his consultation with me.

Mr. W——, a native of the interior of New York, moved to Wisconsin some fifteen years since; pursued an active life as an agriculturalist, often engaging in the favorite sport of hunting and fishing. He was a man of talent and education, and acquired much distinction and influence in the community. A short time before his death, which was caused by tubercular phthisis, demonstrated by a *post-mortem* examination, I was requested to visit him, and learned that for ten years, at least, he had made almost daily use of distilled spirits.

Mr. A——, a native of New York, aged 24 years; married and moved to Chicago, only two years since; has enjoyed good health until eleven months since, when he began to cough, and all the symptoms and signs of tubercular phthisis have been successively developed, until at present he is in the last stage of the disease. From himself and wife I learned that for the last four years he had made daily use of alcoholic beverages; at first moderately, but for the last twelve months to such an extent as to occasionally produce intoxication.

Mrs. G——, a native of Ireland, aged twenty-three years, came to this country two years since, was married and gave birth to a child, which was four months old when she came under my observation.

She was then in the advanced stage of tubercular phthisis, and died one month afterwards. I learned from her, that until the last eighteen months she considered herself in good health, and did not know that there was any hereditary tendency to phthisis in the family to which she belonged. That for three years previous to leaving for America, she had lived as a servant girl with an English family, and had been furnished as regularly with her *mug* of *Beer* as with her meals. Since her arrival in America she had continued to use the Beer, and occasionally a glass of distilled spirits.

Mr. W——, a young man, had been employed as clerk in a saloon, came to me on account of a cough which had been increasing during the previous three months. On examination, found he had lost flesh, had frequent night sweats, some pus in the expectoration, with all the physical signs of tubercular deposits in the middle and upper lobes of one lung in the second stage of its advancement. He said he had seldom tasted of distilled spirits, but had drank from one to four glasses of ale, daily during the last four years.

Mr. G——, a native of Ireland, aged twenty-one years, came under my observation in the early part of last winter. He had just returned from New-Orleans, and was in the last stage of phthisis. He went to New-Orleans three years previous in good health, had resided there during the three years working as a ship-corker. Told me that his daily practice had been, during the whole time there, to drink a glass of some kind of liquor, generally whiskey, at each meal time, and often a fourth glass at lunch.

I thus briefly state a few of the cases, as samples of nearly all of the 68, included in the first class. They were all cases showing that the tubercular disease was developed and regularly progressed through all its stages, in *one third* of the whole number of cases in subjects who were at the time, and had been for from one to twelve years previously, regularly and habitually using either fermented or distilled drinks. In but little less than one half of the whole number of cases, the disease was developed while the subjects of it were only occasionally using these drinks. While in less than *one quarter* of the whole number, the disease was developed in subjects who had for *years* totally abstained from all such drinks. Hence, so far as these personal observations bear upon the subject, they point directly to the conclusion, that the use of alcoholic beverages, however uniform their administration, and however long continued, does not prevent the development of tubercular phthisis. If we turn from the narrow circle of personal observation to more general inquiries we shall be forced to the same conclusion.

Thus by the sickness and mortality reports of the English and American armies, it is made apparent that soldiers who use regular rations of alcoholic drinks, furnish full as high a ratio of deaths from tubercular disease, as any equal number of men who do not use such drinks. Again, the statistics compiled by Dr. Bell of New-York, in his prize Essay on this subject, led him to the conclusion, not only, that the use of alcoholic drinks did not prevent the development of tubercular disease, but that it actually favored it. The second part of the paper briefly presents those experimental inquiries into the positive action of alcohol on the properties and functions of the human system, which were commenced by me in 1850, comparing them with similar inquiries instituted by others both in this country and Europe; and by which it is clearly demonstrated that the presence of alcohol in the system diminishes the great functions of respiration, capillary circulation, calorification, and metamorphosis of tissues; and as a necessary consequence, leads to diminished excretion and the accumulation of effete matter both in the blood and the tissues. Finally, both the general and special causes that are supposed to favor the development of phthisis, such as a damp atmosphere, insufficient muscular exercise, confinement within doors without sufficient ventilation, &c., are brought under review and their modus operandi examined. All these causes are shown to act in one and the same direction, viz: to retard the metamorphosis of tissue and the excretory functions of the lungs, skin, kidneys, &c.

It is thus made to appear, that the causes which are generally acknowledged to favor the development of tubercular consumption, actually produce a pathological condition of the structures and functions of the body, closely analogous to that induced by alcoholic drinks.

This analogy is further shown by the fact, that a large proportion of cases of phthisis are accompanied by fatty degenerations of the liver, kidneys, &c., strikingly similar to the fatty degenerations of the same organs, induced by the protracted use of alcoholic drinks.

From the whole field of inquiry passed over, the following conclusions are deduced, to which the critical attention of the section on Practical Medicine is respectfully invited:

1st. That the development of tubercular diseases, is facilitated by all those agents and influences, whether climatic or hygienic, which directly or indirectly, impair or retard the metamorphosis of the organized structures and the efficiency of the excretory functions.

2d. That both observations and carefully devised experiments show that the presence of alcohol in the human sytem, notwithstanding its temporary exhileration of the nervous system, positively retards both metamorphosis and elimination.

3d. That neither the action of alcohol on the functions of the human body, nor the actual results of experience, furnish any evidence that alcoholic beverages are capable of either preventing or retarding the development of tubercular phthisis.

